# Cone beam computed tomography with oral contrast for accurate diagnosis and surgical planning of pharyngeal leakage and fistula: a case series

**DOI:** 10.1016/j.bjorl.2021.08.001

**Published:** 2021-10-13

**Authors:** Mitsuyoshi Imaizumi, Toshihiko Suzuki, Shigeyuki Murono

**Affiliations:** Fukushima Medical University, School of Medicine, Department of Otolaryngology, Fukushima, Japan

**Keywords:** Cone beam computed tomography with oral contrast (contrast CBCT), Videofluoroscopy, Pharyngeal leakage and fistula, Three-dimensional imaging

## Abstract

**Objective:**

Pharyngocutaneous fistula is one of the severe complications related to head and neck surgeries. Detecting the accurate three-dimensional location of both the fistula and leakage is essential for surgical treatment. Videofluoroscopy is usually used for locating these; however, its imaging is two-dimensional. We evaluated pharyngeal leakage and fistulae using Cone Beam Computed Tomography (CBCT), known for its three-dimensional high spatial resolution imaging, taken in a sitting position, with oral contrast (contrast CBCT).

**Methods:**

Pharyngeal leakage and fistulae were evaluated in a total of 31 subjects by sequentially performing videofluoroscopy and contrast CBCT. The detection accuracy of videofluoroscopy and contrast CBCT for leakage and fistula, as well as the ability to determine the extent and depth for surgical planning, were investigated and compared.

**Results:**

Videofluoroscopy and contrast CBCT showed suspicious leakage and/or fistula in six and three of the 31 subjects, respectively. Surgical findings revealed the presence of leakage and/or fistula in three of the 31 subjects. The positive predictive values of videofluoroscopy and contrast CBCT were 50% (3/6) and 100% (3/3), respectively. Contrast CBCT provided more precise images, showing the extent and depth of leakage and fistula in three-dimensions.

**Conclusion:**

The present study’s results indicate the usefulness of contrast CBCT in terms of accurate diagnosis of leakage and fistula, due to its three-dimensional imaging being performed with the patient in a sitting position.

**Level of evidence:**

4.

## Highlights

• We evaluated pharyngeal leakage and fistulae using contrast CBCT.
• Contrast CBCT provides three-dimensional images taken in a sitting position.
• Contrast CBCT is useful for accurate diagnosis of leakage and fistula.
• Contrast CBCT may be an effective option to detect pharyngeal leakage and fistulae.

## Introduction

Pharyngocutaneous fistula, defined as a dehiscence of the closure of the pharyngeal mucosa, resulting in leakage of saliva and communication with the skin,^1^ is a common and well-known complication related to head and neck surgical procedures, such as laryngectomy, pharyngeal reconstruction after laryngopharyngectomy, and laryngotracheal separation.^2^, ^3^ The reported incidence of pharyngocutaneous fistula following laryngectomy ranged from 5% to 65% in the 1970s and 1980s, and from 9% to 25% in the last decade. Its occurrence vastly increases the length of hospital stay and, consequently, treatment costs.^4^, ^5^ Even minor salivary leakage from the anastomosis can cause serious infection, and requires careful observations, conservative management and/or surgical treatments. Moreover, according to a recent review, conservative therapy was not effective in 10–44% of patients with head and neck fistulae.^6^ Therefore, precisely detecting the presence or absence of leakage and accurately locating the fistula and leakage three-dimensionally are essential for appropriate surgical planning. Videofluoroscopy is usually used for detection of fistulae and leakages,^7^, ^8^ however, its imaging is two-dimensional, which might be insufficient for accurate location, thus hindering effective surgical treatment.

To observe the three-dimensional pharyngeal leakage, conventional Computed Tomography (CT) is typically used. However, to perform cervical CT after the contrast material is swallowed in a sitting position, 5–10 minutes are required to: secure the patient’s head and body in a supine position; determine the scan range; perform test scanning; and confirm the range. During this period, the contrast material can be cleared or decreased by spontaneous swallowing, as spontaneous saliva swallowing is reported to occur at least every minute.^9^ This time lag may lead to the overlooking of minor and/or complicated leakage, or inaccurate localization of fistulae.

On the other hand, Cone Beam Computed Tomography (CBCT) imaging, initially used for dental radiology and known for its three-dimensional high spatial resolution imaging at low radiation dose for evaluation of bony structures in the head and neck, is performed with the patient in a sitting position.^10^, ^11^ However, it has been reported that the soft-tissue resolution of CBCT is not good, leading to a lack of diagnostic information.^10^

To solve the above-mentioned problems, we hypothesized that if the three-dimensional location of the fistula and leakage using CBCT with oral contrast (contrast CBCT) for the improvement of the CBCT resolution could be precisely detected with the patient in a sitting position, it would yield more benefits than the conventional videofluoroscopy and/or cervical CT, such as improved detection accuracy, shorter evaluation time, efficient surgical treatment, wider application to patients, and possibly reduced radiation dose.

We here investigated the usefulness of the application of contrast CBCT for accurate diagnosis and surgical planning of pharyngeal leakage and fistula compared to videofluoroscopy.

## Methods

This study was approved by the Ethics committee of Fukushima University Hospital (2019–154), which is guided by local policy, national laws, and the World Medical Association Declaration of Helsinki.

### Study subjects

The study subjects underwent videofluoroscopy and contrast CBCT sequentially between November 2015 and March 2020 at Fukushima University Hospital. The inclusion criteria were patients who: had undergone videofluoroscopy after head and/or neck surgery, such as laryngectomy, pharyngeal reconstruction or laryngotracheal separation for evaluation of pharyngeal leakage or fistula; and were able to undergo both sequential videofluoroscopy and contrast CBCT in a total of 30 minutes. The exclusion criteria were patients who: were not able to remain in a sitting position for a minimum of 18 seconds; and/or had reduced cognition. Written informed consent for sequential videofluoroscopy and contrast CBCT was obtained from all patients and/or their family members.

Pharyngeal leakage and fistulae were evaluated in a total of 31 subjects. The patient characteristics are described in Table 1, Table 2. Twenty-nine patients were male and two were female. The mean age was 71, and the median age was 72 years (range, 55–83 years) at the time of examination. Diseases included hypopharyngeal cancer (n = 13), laryngeal cancer (n = 11) and aspiration pneumonia (n = 7). Head and neck surgeries included pharyngeal reconstruction after laryngopharyngectomy (n = 14), laryngectomy (n = 10) and laryngotracheal separation (n = 7).

**Table 1:** Patient characteristics.

		No of patients
**Patients**		31
**Age, years**	Mean	71
	Median (range)	72 (55–83)
**Sex**	Male	29
	Female	2
**Diseases**		
Hypopharyngeal cancer		13
Laryngeal cancer		11
Aspiration pneumonia		7
**Surgeries**		
Laryngopharyngectomy		14
Laryngectomy		10
Laryngotracheal separation		7

**Table 2:** Oncologic patient characteristics.

	No of patients
**Oncologic patients**	24
Hypopharyngeal cancer (TNM stage and preoperative treatment if applicable)	13
1. T2N1M0 Radiotherapy + Chemotherapy	
2. T2N2bM0 N/A	
3. T4aN0M0 N/A	
4. T2N2bM0 N/A	
5. T2N2aM0 N/A	
6. T4aN1M0 N/A	
7. T2N2cM0 N/A	
8. T4aN3aM0 N/A	
9. T4aN3bM0 N/A	
10. T2N0M0 N/A	
11. T4aN2bM0 N/A	
12. T4aN2bM0 N/A	
13. T2N2cM0 N/A	
Laryngeal cancer (TNM stage and preoperative treatment if applicable)	11
1. T2N0M0 Radiotherapy	
2. T4aN2cM0 N/A	
3. T3N0M0 N/A	
4. T4aN0M0 N/A	
5. T3N2bM0 N/A	
6. T3N1M0 N/A	
7. T3N0M0 N/A	
8. T3N0M0 N/A	
9. T4aN0M0 N/A	
10. T4aN0M0 N/A	
11. T3N1M0 N/A	

### Videofluoroscopy and contrast CBCT

All participants underwent one videofluoroscopy and one contrast CBCT examination, which were performed and recorded sequentially. The pharyngeal leakage and fistula were imaged with videofluoroscopy (Sonial vision Safire II; Shimadzu Medical Systems, Osaka, Japan) and contrast CBCT (3D Accuitomo; Morita, Kyoto, Japan) using 3 mA, 90 kV, 3.1 mm Al total filtration, 0.5 × 0.5 mm fixed focal spot, and a field of view of 100 mm in height and 140 mm in diameter. The exposure time of CBCT was 17.5s, during which approximately 500 frames of two-dimensional projections (around 750 × 950 pixels) are recorded as the C-arm rotates 360°. The X-Ray detector of CBCT was a flat panel detector with an imaging area of around 200 mm in height and 250 mm in width. The voxel size was 0.125 mm, and the slice thickness used in image review was 0.750 mm. The CTDI_vol_ (volume CT dose index) was 5.8 mGy with a Polymethyl Methacrylate (PMMA) cylindrical phantom of diameter 16 cm.

Ten-ml of oral contrast material (diatrizoate sodium, Gastrografin; Bayer, Osaka, Japan) was administered just before conducting CBCT. After the patient swallowed the material, contrast CBCT was performed with the patient in a sitting position as a static condition. To assess reliability, two head and neck surgeons and one radiologist performed all of the videofluoroscopy and contrast CBCT examinations. Each examination video and image was interpreted by two head and neck surgeons.

## Results

### Detection of leakage and fistula

We determined presence of pharyngeal leakage and fistula by surgical findings at the operating room, and/or infection that required wound care or packing. All examinations were performed safely and smoothly in 31 subjects. Videofluoroscopy and contrast CBCT showed suspicious leakage and/or fistula in six and three subjects, respectively, and the presence of leakage and/or fistula was observed in three patients, as shown in Table 3. Videofluoroscopy had 100% (3/3) sensitivity and 89.2% (25/28) specificity. Contrast CBCT had both a sensitivity and a specificity of 100% (3/3 and 28/28). The positive predictive values of videofluoroscopy and contrast CBCT were 50% (3/6) and 100% (3/3), respectively.

**Table 3:** Characteristics of patients with suspicious leakage.

Case	Age, year	Sex	Disease	Comorbidities	Surgeries	Finding of videofluoroscopy	Finding of CBCT	Finding of surgery	Start date of having food after the examination, day
1	55	M	Laryngeal cancer	–	Laryngectomy	Leakage and fistula	Leakage and fistula	Leakage and fistula	59
2	69	M	Laryngeal cancer	Diabetes *mellitus*	Laryngectomy	Leakage and fistula	Leakage and fistula	Leakage and fistula	13
3	65	M	Hypopharyngeal cancer	Nasal cavity cancer	Laryngopharyngectomy	Leakage and fistula	Leakage and fistula	Leakage and fistula	35
4	75	M	Aspiration pneumonia	Hypertension, COPD	Laryngotracheal separation	Suspicious of leakage	No leakage	N/A	1
5	76	M	Aspiration pneumonia	Esophageal cancer	Laryngotracheal separation	Suspicious of leakage	No leakage	N/A	5
6	81	M	Hypopharyngeal cancer	Atrial fibrillation	Laryngopharyngectomy	Suspicious of leakage	No leakage	N/A	0

### Case examples

Notable patient accounts highlighting precise images, showing the three-dimensional extent and depth of each patient’s leakage and fistula, are highlighted here for select patients.

**Figure 1:**
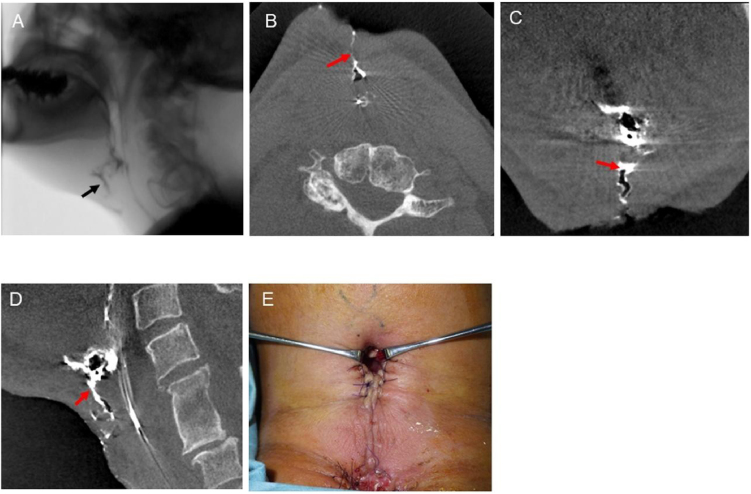
Videofluoroscopy and contrast CBCT findings of Case 1. A, Videofluoroscopy showing the leakage of contrast fluid. The black arrow indicates the leakage. (B–D) Axial, coronal, and sagittal images of contrast CBCT showing the accurate location of the contrast fluid. The red arrows indicate the leakage. E, Surgical treatment of pharyngocutaneous fistula was performed.

**Figure 2:**
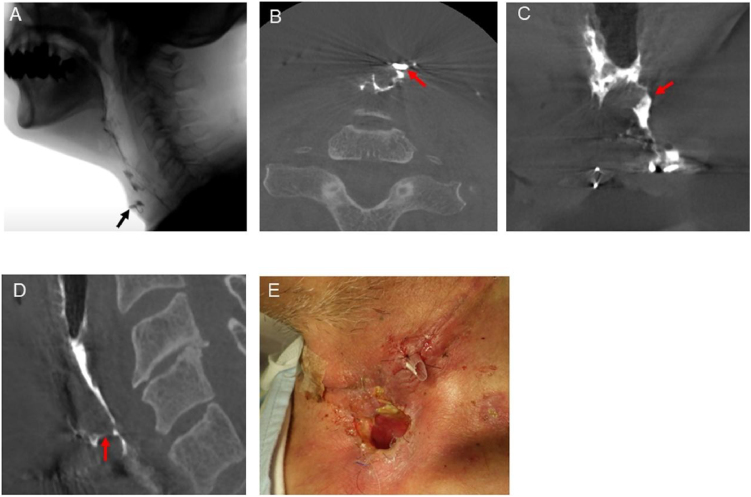
Videofluoroscopy and contrast CBCT findings of Case 2. A, Videofluoroscopy showing the leakage of contrast fluid. The black arrow indicates the leakage. B, Axial image of contrast CBCT showing the accurate location of leakage on the cervical side. C, Coronal image of contrast CBCT showing the accurate location of leakage on the pharyngeal side, which was located not at the midline, but on the left side. D, Sagittal image of contrast CBCT detected another leakage, which could not be observed by videofluoroscopy. The red arrows indicate the leakage. E, Appropriate surgical treatment was performed.

Case 1 was a 55-year-old male patient who underwent laryngectomy due to rT2N0M0 squamous cell carcinoma of the larynx after radiation therapy. Ten days postoperatively, due to purulent effusion at the surgical site, pharyngeal leakage and fistula were suspected. Videofluoroscopy (Fig. 1A) and contrast CBCT (Fig. 2B–D) (Supplementary Material Online Video 1) showed pharyngeal leakage and fistula. In addition, contrast CBCT revealed detailed images of the three-dimensional extent and depth of the leakage and fistula accurately. On the same day, surgical treatment for pharyngocutaneous fistula was performed (Fig. 1E).

Case 2 was a 69-year-old male patient who underwent laryngectomy and bilateral neck dissection reconstructed with anterolateral thigh flap. Fourteen days postoperatively, due to wound dehiscence and necrosis at the surgical site, pharyngeal leakage and fistula were suspected. Videofluoroscopy (Fig. 2A) and contrast CBCT (Fig. 2B) (Supplementary Material Online Video 2) showed pharyngeal leakage and fistula. Coronal image of contrast CBCT showed the accurate location of the leakage on the pharyngeal side, which was located not at the midline, but on the left side (Fig. 2C). Contrast CBCT further revealed another leakage and fistula, which was not detected by videofluoroscopy (Fig. 2D). On the same day, surgical treatment was performed (Fig. 2E).

**Figure 3:**
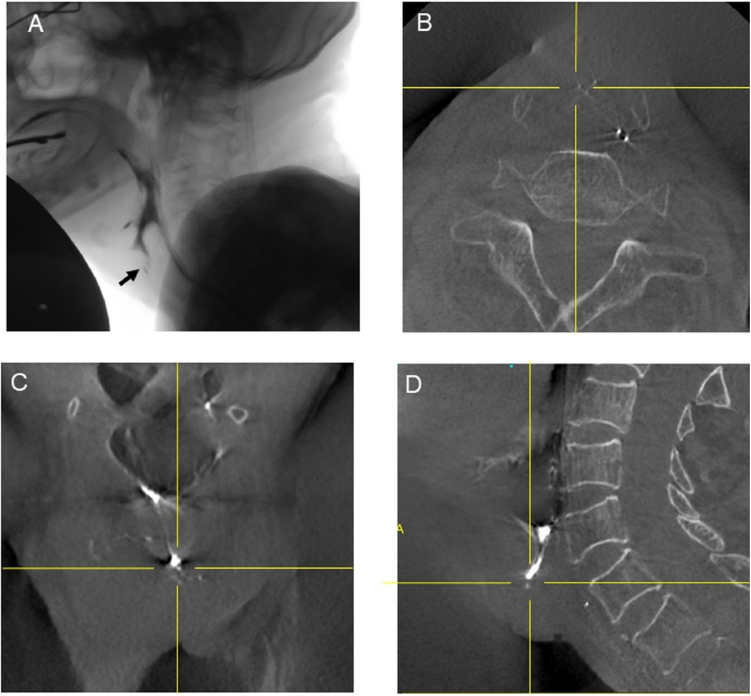
Videofluoroscopy and contrast CBCT findings of Case 4. A, Videofluoroscopy showed a suspicious minor pharyngeal leakage. The black arrow indicates the suspicious leakage. (B–D) The intersection of the vertical and horizontal yellow lines indicates the same location. CBCT detected no leakage.

Case 4 was a 75-year-old male patient who underwent laryngotracheal separation due to intractable aspiration pneumonia. Twenty-one days after surgery, videofluoroscopy showed suspicious pharyngeal leakage (Fig. 3A), whereas contrast CBCT detected no leakage using the combination of three-dimensional, axial, coronal, and sagittal images (Fig. 3B–D). The patient’s recovery was uneventful, and he was discharged as planned.

## Discussion

To the best of our knowledge, the present study is the first to demonstrate the usefulness of contrast CBCT for accurate diagnosis of leakage and fistula and for surgical planning with three-dimensional images, compared to videofluoroscopy. Contrast CBCT may provide sufficient information especially for patients in whom pharyngocutaneous fistula and/or leakage are suspected, and for those who require surgical treatment.

Videofluoroscopy for detection of fistula after head and neck surgery has been reported as a useful examination method.^7^, ^8^ Though videofluoroscopy is an established method, the images produced are two-dimensional. For accurate detection of leakage and fistula, conventional videofluoroscopy is not always sufficient, and three-dimensional images are preferable to two-dimensional images. In terms of obtaining detailed three-dimensional images, conventional CT is typically used for this purpose. However, conventional CT cannot be used to perform an examination on a patient in a sitting position. Recently, in order to observe three-dimensional pharyngeal anatomy and analyze a patient’s swallowing characteristics in detail, the utility of a 320-detector-row Multislice Computed Tomography (MSCT) performed with the patient in a 45°reclining position has been reported.^12^, ^13^ However, swallowed oral contrast material tends to transit along the posterior wall of pharynx in a reclining or lying position; therefore, minor leakage and fistula are more precisely detected or denied using three-dimensional images when the patient is in a sitting position, as demonstrated in the current study.

In general, detection of the cervical side of a fistula is relatively straightforward. However, after opening the wound, we must identify the leakage and fistula three-dimensionally in order to determine the appropriate surgical treatment, without overlooking any possible unopened wounded areas. As shown in Fig. 2, contrast CBCT detected another leakage and fistula which videofluoroscopy could not, by using images of the three-dimensional contrast material-spread space obtained from contrast CBCT images. In addition, it is practically useful to use three-dimensional images of the pharyngeal side of a leakage and its surrounding tissues or organs as shown in Fig. 2C, in order to successfully perform surgery for pharyngocutaneous fistulae. Moreover, the volume flow of the fistula, which was not measured in the present study, from the three-dimensional images taken using contrast CBCT, might provide additional information for surgical planning. As shown in Fig. 3, suspicious minor leakage detected by videofluoroscopy was denied by contrast CBCT using a combination of three-dimensional, axial, coronal, and sagittal images, indicating that the presence or absence of leakage is more precisely detected using contrast CBCT. Therefore, we believe that contrast CBCT may be applicable for not only patients with clinical evidence of fistula for surgical planning, but also patients with suspected fistula.

Videofluoroscopy and CBCT involve the use of X-Rays; therefore, examiners must closely monitor and minimize radiation exposure doses to patients, referring to the “as low as reasonably achievable” principle.^14^, ^15^ We measured the radiation doses of videofluoroscopy, cervical CT and CBCT in the present study. Considering the anatomical location of the thyroid gland in the head and neck, we measured radiation dose at a point 1 cm below the surface of the PMMA cylindrical phantom of diameter 16 cm. Referring to a previous radiation dose study^16^ and the mean screening time of our hospital, we determined the radiation exposure duration during videofluoroscopy to be 5 minutes. The radiation dose of videofluoroscopy was 7.2 mGy/5 minutes, and those of cervical CT and CBCT were 45.1 and 6.0 mGy, respectively. In general, and as shown in our data, the radiation dose of cervical CT is much higher than that of videofluoroscopy and CBCT. Because of the high radiation dose of conventional CT, it is not suitable for repeated examinations for detection of suspicious leakage and fistula; however, CBCT imaging at low radiation doses can be performed repeatedly for this purpose. In addition, the radiation dose of CBCT is lower than that of videofluoroscopy performed in the typical examination time, indicating that for accurate detection of fistula and leakage, as well as for minimizing the radiation dose, contrast CBCT may be an alternative option as far as selecting patients appropriately.

There are several limitations to the current study. First, when contrast CBCT is performed, the subject has to remain in a sitting position for 18 seconds. As we have described in this manuscript, this is one of the important aspects of CBCT. However, depending on their physical condition, sometimes patients cannot sit for that long, and/or can only lie on a bed. Further investigation into how to address this issue with different modalities may be needed in future. Second, the detection accuracy of contrast CBCT was superior to videofluoroscopy; however, videofluoroscopy, with 100% sensitivity, 89.2% specificity and positive predictive values of 50% in the current study, is still useful enough as the first-choice examination for detection of fistulae. Contrast CBCT is an informative examination especially for patients who had suspected leakage and/or fistula found using videofluoroscopy, in order to diagnose the presence or absence of leakage more accurately. There were a limited number of cases available at the time we conducted our study. Further research with larger study populations is necessary to confirm our results.

## Conclusions

The results of the present study indicate the usefulness of contrast CBCT in terms of accurate diagnosis of leakage and fistulae, due to its three-dimensional images, taken with the patient in a sitting position. To detect the presence or absence of leakage more precisely, contrast CBCT, as a low-radiation dose CT, may be an effective option.

## Funding

The authors report no financial interests, relationships, or affiliations relevant to the subject of the manuscript.

## Conflicts of interest

The authors declare no conflicts of interest.

## Acknowledgements

The authors would like to acknowledge Masahiro Harada and Katsumasa Sato for their assistance in measuring radiation dose.

## Appendix A Supplementary data

Online Videos 1 and 2: Rotating 360° view of contrast CBCT images of Cases 1 and 2. Rotating view of the contrast CBCT images showing the accurate three-dimensional location of the fistula and leakage.
